# GABAergic and inflammatory changes in the frontal cortex following neonatal PCP plus isolation rearing, as a dual-hit neurodevelopmental model for schizophrenia

**DOI:** 10.1007/s12035-024-03987-y

**Published:** 2024-02-16

**Authors:** Jennifer A. Cale, Ethan J. Chauhan, Joshua J. Cleaver, Anthoio R. Fusciardi, Sophie McCann, Hannah C. Waters, Juš Žavbi, Madeleine V. King

**Affiliations:** School of Life Sciences, The University of Nottingham, Medical School, Queen’s Medical Centre, Nottingham, NG7 2UH UK

**Keywords:** Neonatal PCP, Isolation rearing, GABA, Parvalbumin, Inflammation, IL-6

## Abstract

The pathogenesis of schizophrenia begins in early neurodevelopment and leads to excitatory-inhibitory imbalance. It is therefore essential that preclinical models used to understand disease, select drug targets and evaluate novel therapeutics encompass similar neurochemical deficits. One approach to improved preclinical modelling incorporates dual-hit neurodevelopmental insults, like neonatal administration of phencyclidine (PCP, to disrupt development of glutamatergic circuitry) then post-weaning isolation (Iso, to mimic adolescent social stress). We recently showed that male Lister-hooded rats exposed to PCP-Iso exhibit reduced hippocampal expression of the GABA interneuron marker calbindin. The current study expanded on this by investigating changes to additional populations of GABAergic interneurons in frontal cortical and hippocampal tissue from the same animals (by immunohistochemistry) as well as levels of GABA itself (via ELISA). Because inflammatory changes are also implicated in schizophrenia, we performed additional immunohistochemical evaluations of Iba-1 positive microglia as well as ELISA analysis of IL-6 in the same brain regions. Single-hit isolation-reared and dual-hit PCP-Iso rats both showed reduced parvalbumin immunoreactivity in the prelimbic/infralimbic region of the frontal cortex. However, this was more widespread in PCP-Iso, extending to the medial/ventral and lateral/dorsolateral orbitofrontal cortices. Loss of GABAergic markers was accompanied by increased microglial activation in the medial/ventral orbitofrontal cortices of PCP-Iso, together with frontal cortical IL-6 elevations not seen following single-hit isolation rearing. These findings enhance the face validity of PCP-Iso, and we advocate the use of this preclinical model for future evaluation of novel therapeutics—especially those designed to normalise excitatory-inhibitory imbalance or reduce neuroinflammation.

## Introduction

Current antipsychotics address the positive symptoms of schizophrenia in approximately 70% of patients but have limited effect on cognitive or negative symptoms. As a result, this disorder remains one of the top 10% causes of disability worldwide, with an approximate cost of $281.6 billion in the USA alone during 2020 and an average lifetime cost of $3.8 million per patient [1]. There have been extensive efforts to develop improved treatments acting via a diverse array of pharmacological targets, but unfortunately, most of those that showed promising activity in preclinical tests failed to progress beyond phase III clinical trials. This high attrition reinforces the need for improved preclinical models, to further elucidate disease neurobiology, select plausible new targets for drug development and enable more predictive evaluation of novel therapeutics [2].

The pathogenesis of schizophrenia begins in early neurodevelopment and leads to lasting excitatory-inhibitory imbalance [3]. There is post-mortem evidence that this is due, at least in part, to disruption of certain populations of GABAergic interneurons in the prefrontal cortex (PFC) and hippocampus. For example, in schizophrenia, these regions contain lower levels of mRNA encoding parvalbumin and somatostatin [4–8], which are markers for respective interneuron subgroups that predominantly target either the cell body and initial axon segment, or dendritic shafts and spines of pyramidal neurons. This is also apparent at the protein level, with patient samples showing fewer parvalbumin- and somatostatin-immunoreactive neurons [5, 9, 10], or reduced intensity of parvalbumin immunostaining [11]. Neuroinflammation has been proposed as a potential mechanism underlying these changes, since positron emission tomography (PET) and post-mortem immunohistochemistry studies in schizophrenia detect increased activation of the brains immune cells, microglia [12]. There are reports of increased microglial cell density in studies examining the major histocompatibility complex class II antigen HLA-DR [13–15], as well as a shift towards a more activated amoeboid morphology of cells labelled for ionised calcium–binding adaptor molecule 1 (Iba-1) [16]. These are accompanied by elevated levels of cytokines, including interleukin-6 (IL-6) [15, 17]. Regardless of whether this inflammation is a cause or consequence of interneuron dysfunction, it is desirable that preclinical models for schizophrenia should feature similar GABAergic and inflammatory changes. This is a realistic expectation in genetic [18] and neurodevelopmental models [19] but cannot be achieved with simple acute pharmacological manipulations.

One approach to producing more comprehensive preclinical models involves ‘dual-hit’ combinations of established perinatal and peripubertal interventions. The two ‘hits’ are each chosen to mirror different aspects of the delayed symptom onset and multiple neurotransmitter involvement characteristic of complex neurodevelopmental disorders like schizophrenia [20]. For example, neonatal NMDA receptor antagonist administration (between postnatal days 7 and 11 when sensitivity to their pro-apoptotic effects peaks [21]) followed by post-weaning isolation rearing of gregarious rat pups induces a more robust phenotype than either manipulation alone [22–25]. Particular advantages of combining neonatal phencyclidine (PCP) with isolation rearing (PCP-Iso) are more extensive cognitive impairment across a broader array of domains (including spatial reference and fear-motivated associative memory [22, 25]), plus altered social interaction and concomitant ultrasonic vocalizations [26, 27] that appear more akin to negative symptomatology than the increased aggression seen with single-hit isolation rearing [28]. These are accompanied by downregulation of hippocampal genes involved in glutamate metabolism, dopaminergic neurotransmission, and GABA receptor signalling, as well as those encoding parvalbumin and glutamic acid decarboxylase 67 (GAD_67_) [29]. PCP-Iso also have reduced hippocampal expression of the calcium-binding protein calbindin [30], which is present in subsets of GABAergic interneurons throughout strata oriens, radiatum and lacunosum-moleculare (where our counts were obtained) as well as glutamatergic cells within stratum pyramidale. This appears consistent with reduced numbers or a disordered pattern of calbindin-positive cells in schizophrenia [31, 32], and may contribute to the apparent improved predictive validity of PCP-Iso compared to single-hit counterparts [30]. To provide further insight, the current study investigated changes to additional parvalbumin- and somatostatin-positive populations of GABAergic interneurons in sub-regions of the frontal cortex and hippocampus plus calbindin-positive cells in the frontal cortex (by immunohistochemistry). We performed additional immunohistochemical evaluation of Iba-1-positive microglia, as well as ELISA analysis of IL-6 and levels of GABA itself in bulk tissue from the same regions. Findings provide an important backdrop against which to interpret neurochemical substrates of the accompanying visual recognition memory deficits [30] in the same animals.

## Methods

### Animals and experimental design

All procedures were conducted in accordance with the Animals (Scientific Procedures) Act, 1986, with approval from the University of Nottingham Animal Welfare and Ethical Review Body (AWERB). The research was designed and is reported in accordance with the Animal Research: Reporting of In Vivo Experiments (ARRIVE) guidelines [33]. It used stored brain tissue from a previously described cohort of 42 male Lister-hooded rats, in which the PCP-Iso group exhibited reduced hippocampal calbindin expression [30].

In summary, rats from a total of 6 litters were obtained with dams on postnatal day (PND) 3 (Charles River UK). They were maintained under controlled conditions throughout the study (21 ± 2 °C, 55 ± 10% humidity, 12-h light–dark cycle; on at 07:00 h) with unlimited access to food and water. Upon arrival, family groups were housed in individually ventilated cages (GR1800 Double-Decker; Tecniplast) containing sawdust bedding and standard environmental enrichment (cardboard play tube, wooden chew block and paper nest material). One pup died between delivery and the start of the study, and the remaining 41 pups, who each represented a single experimental unit, were randomised (by drawing lots) to receive neonatal administration of saline vehicle (Veh; 1 mL/kg s.c.) or PCP HCl (10 mg/kg base; Sigma-Aldrich) on PND 7, 9 and 11. Although this ensured each family group included a mix of both vehicle- and PCP-treated pups (which is an important design consideration to avoid any possibility of litter effects confounding the resulting data) it does introduce the possibility for cross-contamination (as a result of dams ingesting PCP during licking and grooming of drug-treated offspring and passing it to their suckling control offspring via the milk). It is reported that adult rats excrete 93% of an i.v. PCP dose via the urine and faeces within a week [34], but we do not have comparable data for neonatal animals following s.c. administration, nor any indication of milk levels in lactating females following p.o. administration. However, there were no more than three PCP-treated pups in any one litter, and their average cumulative body weights across the dosing period (128.1 ± 16.59 g) together with the pharmacokinetic information outlined above suggest it is possible that each dam had access to in the region of 1.19 mg of eliminated PCP over an 11 day period. Elimination of 93% of this by the routes outlined above leaves a maximum of 7% (0.0833 mg) available for incorporation into milk, and equal division between pups (6.83 per litter) results in a maximal possible cumulative p.o. dose of approximately 0.67 mg/kg to each individual. The dose ‘vehicle-treated’ pups might experience through this unavoidable cross-contamination therefore represents approximately 2% of that likely to encountered by PCP-treated pups through their combination of direct plus indirect routes, and the long-term consequences of this are likely to be minimal. 

At weaning age (PND 21), the Veh-treated rats were further randomised (again by drawing lots) to rearing in standard groups of three or four per cage (Veh-Gr control; *n* = 14) or isolation, i.e. one per cage (single-hit Veh-Iso; *n* = 13). PCP-treated rats were all isolated (PCP-Iso dual hit; *n* = 14). Our study did not include a single-hit PCP-Gr condition because we have already shown that these animals do not exhibit cognitive dysfunction or lasting excitatory-inhibitory imbalance [25, 30]. Our focus here was to further understand the differences between Veh-Iso and PCP-Iso rather than their absence in PCP-Gr. The present approach allowed us to reduce total animal use by 25% and thereby comply with the reduction component of the 3Rs principle (replacement, reduction and refinement). Group sizes were based on previous studies employing the same techniques [24–26]. Following weaning, rats were housed in cages (Gr: 32 × 51 cm, Iso: 25 × 42 cm) containing sawdust bedding without environmental enrichment, and which had grid lids to ensure maintenance of visual, olfactory, and auditory contact between isolation-reared rats and other group- and singly housed rats within the same holding room [35]. Handling was restricted to a single weekly cage change and body weight measurement until behavioural testing.

To assess differences in pharmacological reversal of novel object discrimination (NOD) deficits between Veh-Iso and PCP-Iso, rats underwent NOD (as described in detail elsewhere [30]) on three separate occasions at 1–2 week intervals (PND 57–80). They received an acute i.p. administration of 0.5% methylcellulose 1% Tween-80 vehicle (1 mL/kg), the 5-HT_6_ receptor antagonist SB-399885 (10 mg/kg; Sigma-Aldrich) or mGlu_7_ antagonist MMPIP (10 mg/kg; Tocris), on separate test days, using a cross-over design and in a pseudorandom order. The humane endpoint would have been euthanasia of any rat experiencing a decrease in body weight (up to a maximum permitted limit of − 20%) and/or signs of poor body condition (e.g. piloerection, hunched posture, absence of grooming) although in practice none of these were encountered. Rats were killed by concussion and immediate decapitation on PND 79–80, straight after the final NOD test. The frontal cortex and hippocampus from one hemisphere were dissected on a refrigerated table (4 °C), weighed, frozen in liquid nitrogen and stored at − 80 °C for use in ELISAs. The remaining intact hemisphere was immerse fixed in 4% paraformaldehyde and cryopreserved in 30% sucrose (each overnight at 4 °C) then frozen in isopentane on dry ice and stored at − 80 °C for use in immunohistochemistry. Full blinding of experimenters to neurodevelopmental history throughout the 8–9 weeks of post-weaning housing was not possible due to the obvious visual difference between group and single housing. However, these allocations were concealed throughout tissue processing and analysis.

### Immunohistochemical analysis of parvalbumin, somatostatin, calbindin and Iba-1

Serial coronal sections (60 μm) were obtained throughout the frontal cortex (Bregma 5.20 to 4.00) and dorsal hippocampus (Bregma − 2.56 to − 5.80 [36, 37]), using a freezing microtome (Anglia Scientific). They were stored in antifreeze (30% ethylene glycol; Fisher Scientific, and 30% glycerol; Honeywell, in 0.1 M phosphate buffered saline (PBS); Oxoid) at − 20 °C until free-floating immunohistochemistry. Tissue from one Veh-Gr and one Veh-Iso was excluded due to technical difficulties during slicing, resulting in final group sizes of *n* = 13 Veh-Gr, *n* = 12 Veh-Iso, and *n* = 14 PCP-Iso for immunohistochemistry.

For each brain region, six evenly spaced sections were processed for each of the four selected markers. Sections were washed (4 × 5 min) in PBS to remove antifreeze then incubated (1 h) in 2% normal goat serum (Abcam) in buffer 1 (0.5% bovine serum albumin (BSA); Sigma-Aldrich, 0.3% Triton-X100; Sigma-Aldrich, in PBS) to minimise non-specific binding of the secondary antibody to the tissue. Sections were incubated (overnight, 4 °C) in rabbit polyclonal antibodies against parvalbumin (Abcam ab11427, 1:1000), somatostatin (Abcam ab108456, 1:500), calbindin (Abcam ab108404, 1:500) or Iba-1 (Wako 019–19741, 1:2000), then washed (3 × 5 min) in buffer 2 (0.15% BSA and 0.1% Triton-X100 in PBS) to prevent any unbound primary antibody from interacting with the goat anti-rabbit Alexa-Fluor 568 secondary antibody (Abcam ab175471, 1:500; 1 h in the dark). A series of negative control sections were incubated in primary antibody alone, secondary antibody alone, or buffers only. Sections were washed (2 × 5 min each in buffer 2 then PBS), mounted on gelatinised slides and air-dried. Slides were rinsed with PBS, counterstained with DAPI nuclear stain (Sigma-Aldrich, 1:2000 in dH_2_O; 30 s) rinsed twice with dH_2_O and cover slipped with DABCO fluorescent mounting medium (Sigma-Aldrich; 0.2% in 90% glycerol in PBS) then stored at 4 °C.

**Fig. 1:**
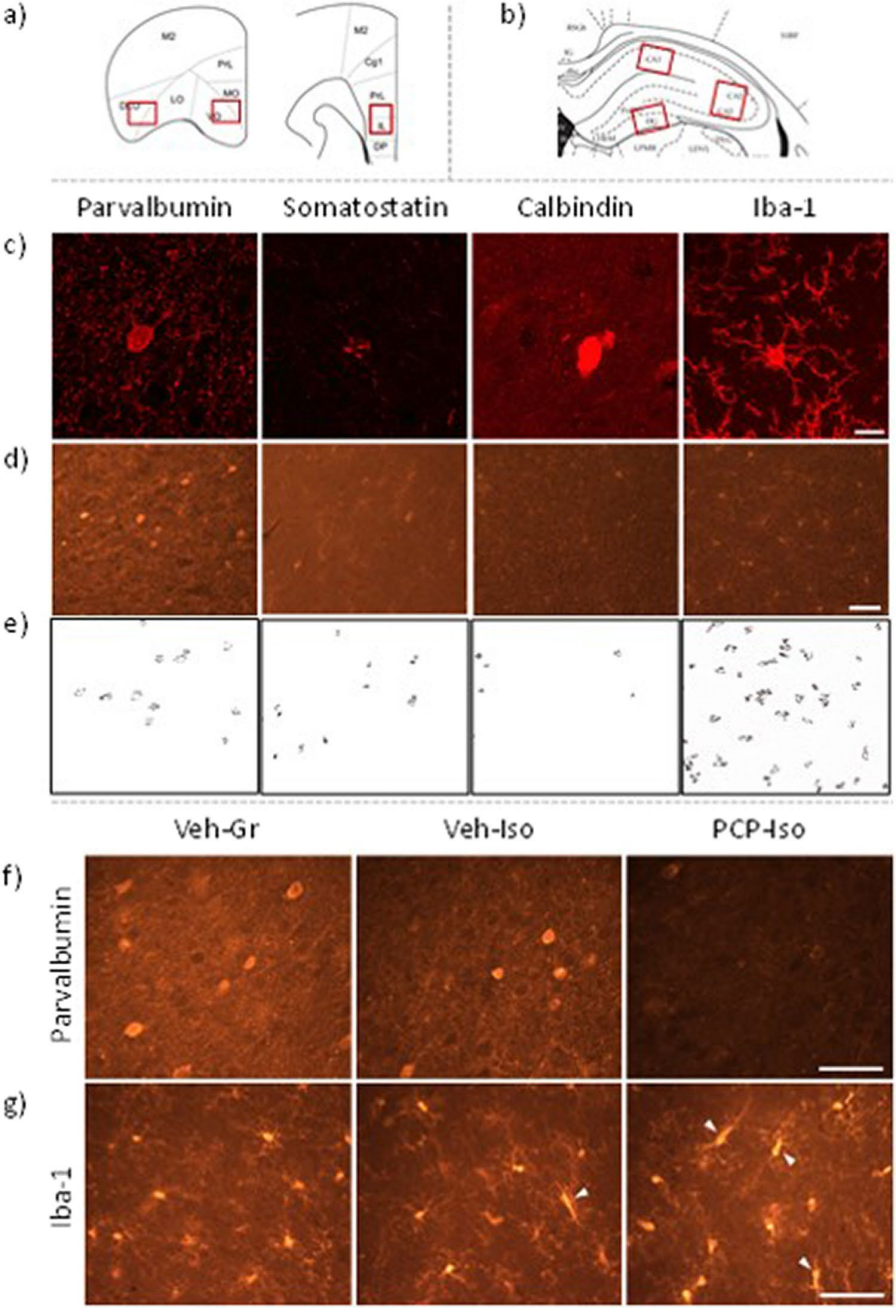
Immunohistochemical staining for GABAergic and inflammatory markers. Consistently placed snapshot images were collected from **a** medial/ventral orbitofrontal (MO/VO), lateral/dorsolateral orbitofrontal (LO/DLO) and prelimbic/infralimbic (PrL/IL) regions of the frontal cortex as well as **b** CA1, CA2/3 and dentate gyrus (DG) subfields of the dorsal hippocampus [36, 37]. Patterns of parvalbumin, somatostatin, calbindin and Iba-1 immunoreactivity in **c** confocal microscopy images obtained from the MO/VO for qualitative insight into the morphology of labelled cells and subcellular localization of the signal, as well as **d** standard fluorescence microscopy images from the MO/VO typical of those used for quantitative analysis. To aid placement of these images within the figure only one quarter of each image is presented. For each marker, **e** features were detected by automated counting settings. Representative higher magnification images from the MO/VO show **f** reduced parvalbumin expression and **g** increased activation state of Iba-1-positive microglia in rats that received PCP on postnatal day (PND) 7, 9 and 11 and were housed in isolation from weaning on PND21 (PCP-Iso), compared to rats that received vehicle injections and were housed in groups (Veh-Gr) or isolation (Veh-Iso). Scale bars are 10 μm in **c** and 100 μm in **d**–**g**. Arrowheads indicate **g** examples of activated microglia with enlarged cell bodies and shortened thickened processes, or transitioning towards a rod-like morphology with narrowed elongated cell bodies and fewer planar processes [41, 42]. Iba-1, ionised calcium–binding adapter molecule 1

To enable qualitative examination of the morphology of immunoreactive cells and subcellular localization of the signal, a small number of control sections were viewed using a Zeiss 880 confocal microscope. Representative × 40 images were obtained using Zen Black software (Zeiss). For quantitative analysis, sections were viewed on a Nikon EFD-3 fluorescence microscope and consistently placed × 10 snapshot images obtained from the medial/ventral orbitofrontal (MO/VO), lateral/dorsolateral orbitofrontal (LO/DLO), and prelimbic/infralimbic (PrL/IL) cortices (Fig. 1a) as well as the CA1, CA2/3, and dentate gyrus (DG) subfields of the hippocampus (Fig. 1b) using a Spot Insight 5MP CM05 USB camera and Spot Advanced software (v5.6; Diagnostic Instruments Inc.). Anatomical boundaries were determined using the stereotaxic brain atlas of Paxinos and Watson and a digital hippocampal atlas [36, 37]. Numbers of parvalbumin-, somatostatin-, calbindin- or Iba-1-positive cells per image were automatically counted with Fiji (Windows 32-bit [38]) by customizing an established protocol [39] to reflect optimal detection settings for each marker. Because it is theoretically possible there might be a decrease in the extent of immunoreactivity per expressing cell without any decrease in the density of immunoreactive cells the intensity of immunoreactivity in each image was automatically determined using the Analyse > Color Histogram tool in Fiji and normalised by subtraction of background staining [40]. In addition, the morphology of each individual Iba-1-positive cell was manually classified to provide an index of activation state. Cells with a small soma and expansive thin processes (whose length > soma diameter) were categorised as resting. Cells with a larger soma and shortened thickened processes (whose length still > soma diameter) as well as those transitioning towards a rod-like shape (narrowed elongated soma with few planar processes) were all classed as activated. Cells with a dramatically enlarged soma and very short or absent processes (whose length ≤ soma diameter) were classed as amoeboid [41, 42]. Data for each rat and brain region were averaged across the six sections per marker, such that n represents the number of biological and not technical replicates.

### ELISA analysis of GABA and IL-6 levels

Frontal cortical and hippocampal samples were homogenised (4 °C) in a 100:1 mix of radioimmunoprecipitation assay (RIPA) buffer and protease inhibitor cocktail (both Sigma-Aldrich). Buffer was added at a ratio of 100μL per 10 mg of tissue and protein extraction achieved by sonication (5–10 s, Soniprep 150; MSE) then vertical disc rotation (1 h). Supernatants resulting from centrifugation (5 min, RCF 850; Eppendorf 5417R) were stored at − 80 °C until analysis. Tissue samples from all animals were included in ELISAs, resulting in final group sizes of *n* = 14 Veh-Gr, *n* = 13 Veh-Iso and *n* = 14 PCP-Iso.

Total protein content was determined by Lowry assay. Forty microliters of BSA standards (0–0.5 mg/mL) and samples (1:100 in dH_2_O) were transferred to a flat bottomed 96-well plate and incubated (10 min) with 40μL of working Lowry reagent (1 part 0.5% copper sulphate pentahydrate, 1 part 2.7% potassium/sodium tartrate and 10 parts sodium carbonate in 0.5 M sodium hydroxide). Folin–Ciocalteu reagent was diluted (1:1 with dH_2_O) and added (20μL) to each well before incubation (45 min, in the dark). Absorbance at 750 nm was read using a SpectraMax M2e plate reader (Molecular Devices) with SoftMap Pro 7.1.2 software.

GABA and IL-6 content was determined against standards using commercially available ELISA kits (Biorbyt orb567856 and orb315058) according to the manufacturer’s instructions. Absorbance at 450 nm was read as described above. GABA and IL-6 concentrations were normalised to total protein concentrations within the same sample to correct for any minor differences in the efficiency of protein extraction between samples.

### Statistical analysis

No exclusion criteria were set and data from all animals were included in the analyses. These were planned before the study took place (although not formally registered) and performed using GraphPad Prism v9.4.1. Normality was assessed with D’Agostino-Pearson or Kolmogorov–Smirnov tests. Based on the outcome of these, intensity of immunoreactivity and ELISA data were analysed by two-way repeated measures ANOVA (with neurodevelopmental condition as a between-subjects factor and brain region or sub-region as a within-subjects factor). The Geisser-Greenhouse correction for unequal variance was applied, and ANOVAs were followed with Tukey’s multiple comparisons post hoc test. Cell density and Iba-1 morphology data were analysed by non-parametric Kruskal–Wallis tests (applied separately to each brain sub-region, with neurodevelopmental condition as the sole factor) followed by Dunn’s post hoc. Data analysed with parametric tests are presented as bar charts showing mean ± standard error of the mean (SEM) and those analysed with non-parametric tests as box and whisker plots showing median, interquartile range (IQR) and 95% confidence intervals (CI). *P* < 0.05 was considered statistically significant.

## Results

### Validation of immunohistochemical staining for GABAergic and inflammatory markers

The selected primary antibodies have been used to visualise parvalbumin [43], somatostatin [44], calbindin [45] and Iba-1 [46] immunoreactivity in rat and mouse brain, and immunostaining in this study is consistent with previously observed patterns in the rat frontal cortex and hippocampus [41, 47, 48]. Thus, parvalbumin, calbindin and Iba-1 immunoreactivity were present throughout nuclei, cell bodies and processes (Fig. 1c–d). Abundant labelling of cell bodies enabled automated counting of immunoreactive cells to be reliably performed (Fig. 1e). Somatostatin immunoreactivity was less intense overall and showed a comparative absence from intracellular regions of the cell body (Fig. 1c–d). As a result, the settings necessary to automatically count cell bodies frequently also detected portions of immunoreactive processes irrespective of whether or not these were from the same or different cells (Fig. 1e). Somatostatin-positive cell counts should therefore be interpreted with caution. We have confidence in remaining data, which is maximised by that fact that immunoreactivity was only observed in sections incubated with respective primary plus the secondary antibody and abolished by the absence of primary and/or secondary antibodies (data not shown).

### Impact of combined neonatal PCP and isolation rearing on GABAergic markers

**Fig. 2:**
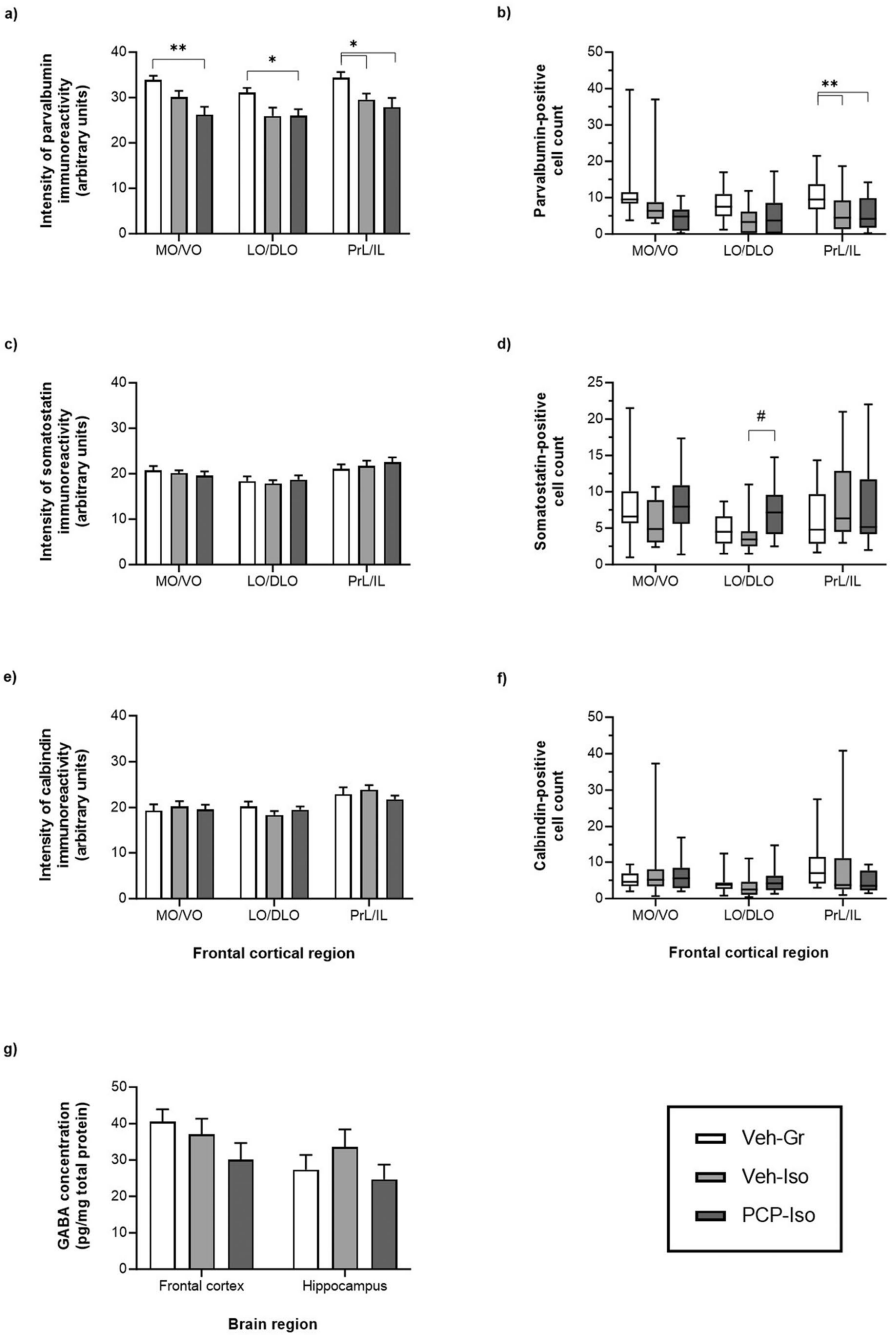
Impact of combined neonatal PCP and isolation rearing on GABAergic markers in the frontal cortex. Mean ± SEM intensity of **a** parvalbumin, **c** somatostatin and **e** calbindin immunoreactivity, together with median, IQR (box) and 95% CI (whiskers) for counts of **b** parvalbumin-, **d** somatostatin- and **f** calbindin-positive cell densities, as well as **g** mean ± SEM levels of GABA itself in both frontal cortical and hippocampal homogenates from opposite hemispheres of the same animals. Male Lister-hooded rats received saline vehicle (1 mL/kg s.c.; Veh) or PCP (10 mg/kg) on postnatal day (PND) 7, 9 and 11 were housed in groups (Gr) or isolation (Iso) from weaning on PND 21. They underwent novel object discrimination (NOD) three separate times at 1–2 week intervals (PND 57–80) following acute i.p. administration of 0.5% methylcellulose 1% Tween-80 vehicle (1 mL/kg), SB-399885 (10 mg/kg) or MMPIP (10 mg/kg) on separate days using a cross-over design. Tissue was collected on PND 79–80 (*n* = 12–14 per neurodevelopmental condition) and immunohistochemical data were obtained from consistently placed regions of interest within medial/ventral orbitofrontal (MO/VO), lateral/dorsolateral orbitofrontal (LO/DLO) and prelimbic/infralimbic (PrL/IL) sub-regions. *P < 0.05; **P < 0.01 Veh-Iso and PCP-Iso versus Veh-Gr; #*P* < 0.05 PCP-Iso versus Veh-Iso (**a**,** c**,** e**,** g** two-way repeated measures ANOVA with Tukey’s or **b**,** d**,** f** Kruskal–Wallis test with Dunn’s post hoc)

There was a main effect of neurodevelopmental condition on parvalbumin immunoreactivity throughout the frontal cortex (F_(2,36)_ = 11.40, *P* = 0.0001). This was reduced in the PrL/IL of both single-hit Veh-Iso and dual-hit PCP-Iso (− 14 and − 19%; *P* < 0.05 versus Veh-Gr control, Fig. 2a). Parvalbumin-positive cell density in the PrL/IL showed a similar effect of neurodevelopmental condition (Kruskal–Wallis statistic = 9.178, *P* = 0.0102) and was also reduced in both Veh-Iso and PCP-Iso (− 56 and − 49%; *P* < 0.05, Fig. 2b). Of note, PCP-Iso showed additional decreases in both the intensity of parvalbumin immunoreactivity in the MO/VO (− 23%; *P* < 0.01) and LO/DLO (− 16%; *P* < 0.05), and in the density of parvalbumin-positive cells in the MO/VO (− 71%; *P* < 0.01), neither of which were significantly reduced in Veh-Iso (Figs. 1f and 2a–b).

There was no effect of neurodevelopmental condition on the intensity of frontal cortical somatostatin immunoreactivity (F_(2,36)_ = 0.056, *P* = 0.9455; Fig. 2c), and although somatostatin-positive cell density in the LO/DLO was influenced by neurodevelopmental condition (Kruskal–Wallis statistic = 8.442, *P* = 0.0147) there was no decrease in Veh-Iso or PCP-Iso compared to Veh-Gr. However density in PCP-Iso was higher than in Veh-Iso (Fig. 2d). As noted above, these data should be interpreted with a degree of caution (see Fig. 1e). There were no differences in calbindin immunoreactivity or cell densities in the frontal cortex (Fig. 2e–f), nor in any of the interneuron markers within the hippocampus (data not shown). Levels of GABA were not significantly impacted by neurodevelopmental condition, although interestingly apparent trends within the frontal cortex (Fig. 2g) mirrored the pattern of reduced parvalbumin immunoreactivity across this region (Fig. 2a–b).

### Impact of combined neonatal PCP and isolation rearing on inflammatory markers

**Fig. 3:**
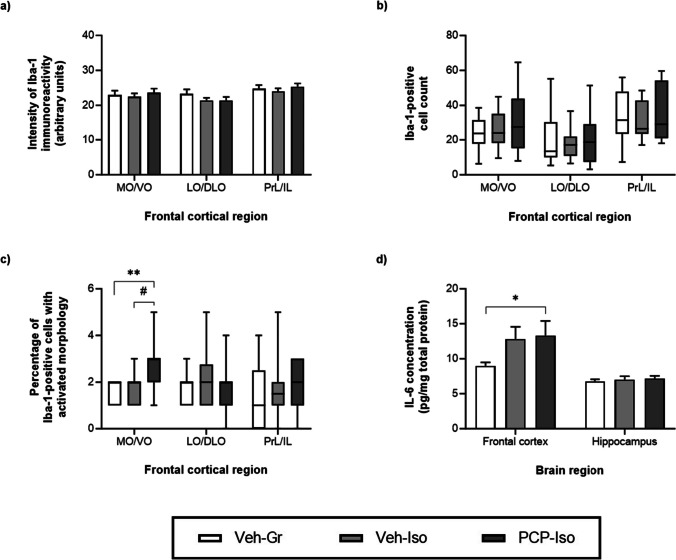
Impact of combined neonatal PCP and isolation rearing on inflammatory markers in the frontal cortex. Mean ± SEM **a** intensity of Iba-1 immunoreactivity, together with median, IQR (box) and 95% CI (whiskers) for **b** Iba-1-positive cell densities and the **c** percentage of Iba-1-positive microglia with morphological indications of an activated state [41, 42], as well as **d** mean ± SEM levels of the cytokine IL-6 in both frontal cortical and hippocampal homogenates from opposite hemispheres of the same animals. Male Lister-hooded rats received saline vehicle (1 mL/kg s.c.; Veh) or PCP (10 mg/kg) on postnatal day (PND) 7, 9 and 11 were housed in groups (Gr) or isolation (Iso) from weaning on PND 21. They underwent novel object discrimination (NOD) three separate times at 1–2 week intervals (PND 57–80) following acute i.p. administration of 0.5% methylcellulose 1% Tween-80 vehicle (1 mL/kg), SB-399885 (10 mg/kg) or MMPIP (10 mg/kg) on separate days using a cross-over design. Tissue was collected on PND 79–80 (*n* = 12–14 per neurodevelopmental condition) and immunohistochemical data were obtained from consistently placed regions of interest within medial/ventral orbitofrontal (MO/VO), lateral/dorsolateral orbitofrontal (LO/DLO) and prelimbic/infralimbic (PrL/IL) sub-regions. *P < 0.05; **P < 0.01 PCP-Iso versus Veh-Gr; #*P* < 0.05 PCP-Iso versus Veh-Iso (**a**,** d** two-way repeated measures ANOVA with Tukey’s or **b**,** c** Kruskal–Wallis test with Dunn’s post hoc)

There was no effect of neurodevelopmental condition on overall expression of Iba-1, either in terms of immunoreactivity or cell densities in the frontal cortex (Fig. 3a–b) or hippocampus (data not shown). However, when Iba-1-positive cells were classified according to their morphology to obtain an index of activation state, there was an effect of neurodevelopmental condition on the percentage of MO/VO cells in the resting state (Kruskal–Wallis statistic = 6.371, *P* = 0.0414), which tended to decrease in PCP-Iso compared to Veh-Gr (0.0797; data not shown). Neurodevelopmental condition also influenced the percentage of MO/VO cells in the early stages of the activated state (Kruskal–Wallis statistic = 13.06, *P* = 0.0015), which was higher in PCP-Iso than both Veh-Gr (1.8-fold; *P* < 0.01) and Veh-Iso (1.6-fold; *P* < 0.05; Figs. 1g and 3c). The percentage of cells in the later, amoeboid stage of the activated state was extremely low (medians ≤ 1%, IQRs 0–1%) and not influenced by neurodevelopmental condition (data not shown). Levels of the inflammatory cytokine IL-6 within the frontal cortex were also higher in PCP-Iso (but not Veh-Iso) than Veh-Gr (*P* < 0.05; Fig. 3d). There were no differences in inflammatory markers within the hippocampus.

## Discussion

Improved preclinical models are essential to enable more predictive evaluation of urgently needed novel therapeutics for negative and cognitive symptoms of schizophrenia. The dual-hit combination of neonatal PCP followed by insolation rearing is reported to produce a wider range of behavioural and neurochemical changes than single-hit isolation rearing, and alterations in PCP-Iso appear more akin to those in schizophrenia [22, 25–27, 30]. The current study is the first to show additional parvalbumin deficits, microglial activation and IL-6 elevation within the frontal cortex of PCP-Iso, and these findings further support improved face validity of the dual-hit PCP-Iso model.

Decreased frontal cortical or hippocampal expression of parvalbumin has been reported following a variety of protocols involving some form of social isolation as a single developmental manipulation. This includes work in highly gregarious rodents like rats, and also studies in mice—despite males of this species generally being more aggressive towards conspecifics [49]. However, the outcomes in these single-hit models are variable in terms of the affected brain region (e.g., [50, 51] versus [52, 53]). Moreover, they are frequently localised to an individual sub-region, like the PrL or IL as observed in our Veh-Iso [24, 54–56], or even to a specific cell layer within a sub-region [57]. This explains why they might not be detected during analysis of pooled sub-regions [58]. To the best of our knowledge, no studies have assessed the impact of either post-weaning isolation rearing or adulthood social isolation on frontal cortical or hippocampal somatostatin expression. Isolation-induced changes to other markers appear more variable. For example, decreased hippocampal calbindin in our Veh-Iso [30] is more localised than in isolation-reared females from the same strain [50]. The current absence of any frontal cortical change to this marker is in keeping with one prior report [24] but conflicts with mixed findings from another group, who have reported both decreases [59] and increases [60]. Similarly, we [40, 61] and others [62, 63] variously detect increases, decreases or no change to levels of cytokines like IL-6 in the frontal cortex of isolates. Taken together, these findings reinforce the need for more robust models featuring a wider and more reproducible spectrum of changes akin to those in complex psychiatric disorders like schizophrenia.

There is growing appreciation for the value of dual-hit neurodevelopmental models [2, 20]. Although not all combinations of relevant maternal, dietary or social risk factors have an additive effect on rodent behavioural or neurochemical endpoints [40, 64, 65], there is now a strong body of evidence that the consequences of post-weaning social isolation are exacerbated by prior blockade of NMDA receptors during the neonatal period [22, 24–27, 30, 52, 66]. Reduced frontal cortical parvalbumin expression in PCP-Iso mirrors a number of post-mortem findings from schizophrenia patients over the past 25 years. This applies to both the dorsolateral PFC [10, 11, 67, 68] (whose closest functional homolog in rodents includes the PrL [69], impacted by both Veh-Iso and PCP-Iso here) and the orbitofrontal cortex [9] (crucially affected only by PCP-Iso). Evidence for reduced hippocampal parvalbumin in schizophrenia is more sparse [5, 70], and negative reports for both regions [71–73] have led to the proposal of a ‘low GABA marker’ subgroup, comprising approximately 50% of the patient population [74]. There is also an inflammatory subgroup [15, 75, 76] and whilst additional research is needed to clarify the proportion of patients exhibiting low GABA markers [74] in conjunction with increased brain regional cytokines [17], we feel PCP-Iso have the potential to provide insights into disease neurobiology and treatment strategies for this combined subgroup. There is ongoing debate whether reduced expression of calcium binding proteins in schizophrenia represents a selective loss of these proteins from the interneurons, or a loss of the cells themselves. Reports of reduced total cortical neuron number and density [77–79] point towards cell loss, yet these are conflicted by reports of unaltered neuronal numbers or density [80, 81]. Similar conflicts exist for studies that have examined mRNA encoding general GABAergic markers like isoforms of the GABA synthesis enzyme GAD [81–84] versus the vesicular GABA transporter (vGAT) [7, 85–87]. However, a recent meta-analysis of proton magnetic resonance spectroscopy in patients points towards lower levels of GABA itself within the PFC [88] and trends within the current PCP-Iso data appear to mimic this. We feel this may be indicative of an interneuron dysfunction that goes beyond a selective absence of parvalbumin from otherwise normal cells. A reduction in calcium binding protein content is proposed to render cells less excitable, due to reduced ability to buffer Ca^2+^ transients and regulate repolarization. This in turn would disinhibit control over pyramidal cell output, with a similar outcome predicted upon loss of parvalbumin-positive cells [89]. However this view may represent an oversimplification since frontal cortical slices from mice subjected to two weeks of social isolation in the critical post-weaning period (PND 21-35) and then allowed to resocialize actually exhibit increased excitability of parvalbumin-positive interneurons in layer V of the PrL [90, 91].

Veh-Iso and PCP-Iso from this cohort both exhibited deficits in visual recognition memory in the NOD task [30], which means this robust phenotype has been reliably observed in each of the nine PCP-Iso cohorts now examined in our laboratory, despite only occurring in 70% of our previous Veh-Iso studies [30, 35]. Localised disruption of parvalbumin-, somatostain- and neuronal nitric oxide synthase (nNOS)–positive interneurons in the hippocampus is sufficient to impair NOD [92], but cognitive deficits in our Veh-Iso and PCP-Iso cannot be attributed to altered parvalbumin, somatostatin or inflammatory marker expression in this region, nor to somatostatin or calbindin alterations in the frontal cortex since no changes were detected. Neurochemical disturbances in PCP-Iso will extend beyond the molecules [29] and brain regions [66] studied here, so we cannot definitively state that frontal cortical parvalbumin, microglial and/or IL-6 alterations contribute to accompanying cognitive changes. The medial PFC (mPFC) and orbitofrontal cortex are actually more strongly implicated in working memory, reversal learning, attentional set shifting and social cognition [93]. Nevertheless, lesions of the PrL and IL profoundly impair performance in a food-motivated object-based working memory test [94], and NOD is attenuated by localised administration of the GABA_A_ receptor agonist muscimol into the mPFC [95]. With regard to parvalbumin-positive neurons in this region, localised knockdown of the calcium-binding protein or selective inhibition of GABA release from these cells reduces spatial working memory, reversal learning [96] and social interaction [97]. And although transient optogenetic inhibition of these neurons does not appear to impact these cognitive processes [98, 99], optogenetic stimulation or chemogenetic activation of parvalbumin-positive cells in the PFC is able to overcome NMDA receptor antagonist–induced impairments in working memory and cognitive flexibility [100, 101]. Whilst these elegant approaches have not been used to examine the role of frontal cortical parvalbumin-positive neurons in NOD, it is intriguing to note that acute inflammation elevates recruitment of these neurons within the PrL during this task [102]. It is therefore conceivable that reduced parvalbumin expression could contribute to NOD impairments, particularly in the face of PCP-Iso–induced inflammation. To provide more definitive insight into the neurochemical substrates of cognitive and social deficits in PCP-Iso future work should examine whether these can be reversed by optogenetic or chemogenetic stimulation of parvalbumin-positive neurons, as demonstrated for aggressive behaviour in a single-hit social isolation model [54].

We acknowledge that some of our methodological choices may have impacted on the findings, and future work should also attend to these considerations. For example, rats were bred at a commercial establishment and shipped to our laboratories as neonates, arriving (PND 3) four days before the start of treatment (PND 7). It is recognised that transportation can have lasting effects on a wide array of physiological and behavioural parameters, including plasma corticosterone [103], and the age-critical nature of our PCP administration regime allows limited scope for an extended acclimatization period while these normalise. Although one potential alternative would be to breed animals in-house to avoid the need for transport, it is conceivable that stress levels at the time of PCP administration and the start of isolation rearing may interact with our intentional interventions and contribute to the final phenotype. Under those circumstances attempted refinements could have negative implications in terms of replication, and a dedicated study would be needed to assess the consequences of such methodological change. We also acknowledge that the current analysis was restricted to male rats, and this decision was originally taken because there are well-recognised biological sex differences in the human illness we seek to model, including worse premorbid functioning, an earlier age at symptom onset and greater severity of negative symptoms in males [104]. Although previous literature shows that cognitive impairment [105] and localised parvalbumin deficits do occur in single-hit isolation-reared female rats [50], emerging findings demonstrate that microglial activation is much more pronounced in isolation-reared male than female mice [106]. We are yet to examine female PCP-Iso but the current findings in male PCP-Iso, together with indications that perhiperal inflammatory biomarkers in patients vary according to biological sex [107] and a general move towards representation of both sexes in preclinical and clinical trials [108], make studies in female PCP-Iso increasingly timely. Lastly, we note that the brain regional samples analysed here were obtained from rats that each received one administration of the 5-HT_6_ receptor antagonist SB-399885, one administration of the mGlu_7_ antagonist MMPIP and one administration of vehicle control prior to cognitive testing in the 3 weeks leading up to tissue collection for immunohistochemistry. It would be highly unlikely for acute treatment to impact on the immunohistochemical data presented here or in our prior manuscript from the same study [30], and treatments were all administered in a pseudorandom order that was fully balanced across Veh-Gr, Veh-Iso and PCP-Iso groups to avoid any potential confounds based on recency.

Atypical antipsychotic agents are able to correct some, though by no means all of the disordered behaviours in male PCP-Iso. Acute administration of aripiprazole or cariprazine reversed locomotor hyperactivity and NOD impairments without marked effects on social interaction and fear memory [27]. Similarly chronic administration of clozapine reversed locomotor hyperactivity and enhanced social recognition memory without normalizing sensorimotor gating or reversal learning [109]. The anticonvulsant lamotrigine, which blocks voltage-gated sodium channels to reduce excitatory neurotransmission, also reversed locomotor hyperactivity and improved NOD without correcting sensorimotor gating [29]. These reports are broadly consistent with the moderate clinical efficacy of these compounds in schizophrenia (e.g. [110–112]), and the model also replicates negative clinical findings with 5-HT_6_ receptor antagonists [30, 113]. Given our new confirmation of inflammation in PCP-Iso an obvious next step in our reverse translational assessments of predictive validity would be to examine an anti-inflammatory agent, since these have also shown modest efficacy in patients [114, 115]. Another key priority, given the current GABAergic findings, is positive modulators of the Kv3.1/3.2 voltage-gated potassium channels on parvalbumin-positive interneurons. These compounds appear to normalise parvalbumin-positive cell counts in alternative models for schizophrenia [116] and have recently shown promising effects on gamma oscillations in patients [117]. In terms of forward translation, chronic administration of a glycine transporter (GlyT1) inhibitor is so far the only manipulation to normalise social interaction and aberrant patterns of prosocial ultrasonic vocalizations in PCP-Iso [26]. This class of compounds have subsequently been shown to restore inhibitory neurotransmission and normalise excitatory-inhibitory imbalance [118], and emerging clinical evidence suggests they have real potential for improved therapeutic effect [119]. Metabotropic glutamate receptor 7 (mGlu_7_) negative allosteric modulators are at an earlier stage in the drug development pipeline [120, 121], and our preliminary demonstration that they reverse NOD deficits in PCP-Iso [30] certainly suggests more in-depth assessment in this model is now warranted. In conclusion, the current demonstration of frontal cortical parvalbumin reduction, microglial activation and cytokine elevation enhance the face validity of our PCP-Iso model, and support its use to further elucidate disease neurobiology and select plausible new targets for drug development. Accumulating evidence supports the predictive validity of the PCP-Iso model and we advocate more widespread adoption of this valuable tool for preclinical evaluation of novel therapeutics for schizophrenia—especially those designed to normalise excitatory-inhibitory imbalance or reduce neuroinflammation.

## Data Availability

The data generated during this study are available from the corresponding author on reasonable request.
